# Statistical Resolution of Ambiguous HLA Typing Data

**DOI:** 10.1371/journal.pcbi.1000016

**Published:** 2008-02-29

**Authors:** Jennifer Listgarten, Zabrina Brumme, Carl Kadie, Gao Xiaojiang, Bruce Walker, Mary Carrington, Philip Goulder, David Heckerman

**Affiliations:** 1 Microsoft Research, Redmond, Washington, United States of America; 2 Partners AIDS Research Center, Massachusetts General Hospital, Harvard Medical School, Boston, Massachusetts, United States of America; 3 SAIC-Frederick, National Cancer Institute, Frederick, Maryland, United States of America; 4 Howard Hughes Medical Institute, Frederick, Maryland, United States of America; 5 Department of Paediatrics, University of Oxford, Oxford, United Kingdom; University of Washington, United States of America

## Abstract

High-resolution HLA typing plays a central role in many areas of immunology, such as in identifying immunogenetic risk factors for disease, in studying how the genomes of pathogens evolve in response to immune selection pressures, and also in vaccine design, where identification of HLA-restricted epitopes may be used to guide the selection of vaccine immunogens. Perhaps one of the most immediate applications is in direct medical decisions concerning the matching of stem cell transplant donors to unrelated recipients. However, high-resolution HLA typing is frequently unavailable due to its high cost or the inability to re-type historical data. In this paper, we introduce and evaluate a method for statistical, in silico refinement of ambiguous and/or low-resolution HLA data. Our method, which requires an independent, high-resolution training data set drawn from the same population as the data to be refined, uses linkage disequilibrium in HLA haplotypes as well as four-digit allele frequency data to probabilistically refine HLA typings. Central to our approach is the use of haplotype inference. We introduce new methodology to this area, improving upon the Expectation-Maximization (EM)-based approaches currently used within the HLA community. Our improvements are achieved by using a parsimonious parameterization for haplotype distributions and by smoothing the maximum likelihood (ML) solution. These improvements make it possible to scale the refinement to a larger number of alleles and loci in a more computationally efficient and stable manner. We also show how to augment our method in order to incorporate ethnicity information (as HLA allele distributions vary widely according to race/ethnicity as well as geographic area), and demonstrate the potential utility of this experimentally. A tool based on our approach is freely available for research purposes at http://microsoft.com/science.

## Author Summary

At the core of the human adaptive immune response is the train-to-kill mechanism in which specialized immune cells are sensitized to recognize small peptides from foreign sources (e.g., from HIV or bacteria). Following this sensitization, these immune cells are then activated to kill other cells which display this same peptide (and which contain this same foreign peptide). However, in order for sensitization and killing to occur, the foreign peptide must be “paired up” with one of the infected person's other specialized immune molecules—an HLA molecule. The way in which peptides interact with these HLA molecules defines if and how an immune response will be generated. There is a huge repertoire of such HLA molecules, with almost no two people having the same set. Furthermore, a person's HLA type can determine their susceptibility to disease, or the success of a transplant, for example. However, obtaining high quality HLA data for patients is often difficult because of the great cost and specialized laboratories required, or because the data are historical and cannot be retyped with modern methods. Therefore, we introduce a statistical model which can make use of existing high-quality HLA data, to infer higher-quality HLA data from lower-quality data.

## Introduction

The Major Histocompatibility Complex (MHC), located on the short arm of chromosome 6, encodes the Human Leukocyte Antigen (HLA) class I and II genes, whose protein products play an essential role in the adaptive immune response. The HLA class I and class II proteins bind antigenic, pathogen-derived peptides (called *epitopes*) and display them on the cell surface for recognition by CD8+ or CD4+ T-lymphocytes, respectively, thus activating the cellular immune response and mediating pathogen clearance. Critically, each HLA protein can bind only a limited range of peptides (as dictated by HLA-specific binding motifs), and individuals express different (and multiple) HLA class I and class II proteins with different peptide specificities. In addition, the HLA class I and II genes represent the most polymorphic set of genes in the human genome; extensive MHC/HLA genetic diversity on both an individual as well as a population level ensures that the human immune response will be equipped to target a diverse range of pathogens. To date, more than 600, 900, and 300 different alleles have been identified, respectively, for the class I HLA-A,-B and -C-loci, whereas more than 600 alleles have been identified at the class II HLA-DRB1 locus; new alleles are routinely being discovered [1]. In addition, due to their location within the MHC region on chromosome 6, HLA alleles are in tight linkage disequilibrium, and thus can be thought of in terms of a haplotype [2].

High-resolution HLA typing (meaning the determination of the specific HLA alleles which an individual expresses at each of the class I and/or class II loci) is an essential tool for basic as well as clinical immunology research. For example, HLA typing has been used to identify immunogenetic risk factors for human diseases [3],[4],[5] and more recently has been used to investigate how pathogens (such as HIV (e.g., [6],[7]) and, more recently Hepatitis C Virus [8],[9],[10]) evolve in response to HLA-restricted immune selective pressures. In addition, HLA typing is essential for vaccine research: the identification and mapping of HLA-restricted T-cell epitopes in the proteomes of different pathogens (e.g., [11]), could help inform the selection of potential immunogens in a T-cell based vaccine design. Clinically, high-resolution HLA typing is routinely required in the context of modern transplantation medicine, such as for hematopoietic stem cell transplants: in order to minimize risk of rejection, donors and unrelated recipients must be matched with respect to HLA alleles expressed [12].

Historically, HLA typing was performed using low-resolution, antibody-based serological tests. However, higher-resolution HLA typing is now achievable using more modern, molecular (DNA-based) methods. Molecular methods for HLA typing include hybridization with sequence-specific oligonucleotide probes (SSOP), PCR amplification with sequence-specific primers (PCR-SSP), and more recently, DNA sequence-based methods. Generally, DNA sequence-based methods involve locus-specific PCR amplification of exons 2 and 3 (for HLA Class I genes), or exon 2 only (for HLA class II), followed by “bulk” DNA sequencing of the amplified product (i.e., sequencing of products derived from both HLA haplotypes). Sequencing is restricted to exons 2 and/or 3 because these regions are the major determinants of HLA peptide-binding specificity and thus contain enough information to discriminate between most allele combinations. If an individual is heterozygous (i.e., possesses two different alleles) at any locus, direct sequencing of an amplified PCR product will yield nucleotide mixtures at positions in which the two alleles differ in sequence. Consequently, there are two reasons why modern sequence-based typing methods may yield ambiguous typing results: first, if the differences between the two alleles are located outside the genotyped region (in most cases, exons 2 and/or 3), and secondly, if two or more allele combinations yield the exact same pattern of heterozygous nucleotide mixtures when combined into a “bulk” sequence.

Because of the great (and ever-increasing) number of HLA alleles (and thus growing list of ambiguous combinations), unambiguous HLA typing is costly, laborious, and limited to laboratories specializing in this work. For the purposes of scientific research, HLA types are not always unambiguously determined; rather, they are only determined up to some “resolution” (i.e., level of ambiguity). Additionally, because the number of HLA alleles is constantly increasing, sequence-based, SSOP and SSP based typing results, which depend on the list of known alleles, require constant re-interpretation in light of newly discovered alleles. This re-interpretation can result in more ambiguity than originally thought [13]. Perhaps even more importantly, it is often impossible to re-type historic samples that may have been typed using lower-resolution approaches.

The practical consequence of these issues is that there is a large incongruence between the high-resolution HLA typing required for scientific investigations and the HLA data that is widely available. As such, any method which can help to increase resolution of HLA data, *post-hoc* and at low cost, will provide a greatly needed service to the scientific and clinical communities. In this paper, we introduce and evaluate a method for statistical, in silico refinement of ambiguous HLA types. Our method uses information available from inferred HLA haplotypes to probabilistically refine HLA data. Our method, which relies upon haplotype inference from unphased data, introduces new methodology to this area which improves upon the most commonly used approach within the HLA community (i.e., multinomial parameterization trained with an EM—Expectation-Maximization—algorithm).

Our improvements are achieved by using a parsimonious parameterization, and by smoothing the maximum likelihood (ML) solution. These improvements make it possible to scale the refinement to a larger number of alleles and loci in a more computationally efficient and stable manner. We also show how to augment our method in order to make use of data arising from different ethnic backgrounds, and show the potential use of this experimentally. Our method is evaluated using data from various sources, and from various ethnicities, as described in the Experimental section. Additionally, an implementation of our method is available for community-wide use.

### HLA Nomenclature and Typing Ambiguity

HLA nomenclature is closely tied to the levels of possible HLA ambiguity. Each HLA allele is assigned a letter (or letters) which designate the locus (e.g., A, B, and C for class I; DRA, DRB1, DRB2-9, DQA1, DQB1, DPA1, DPB1, for class II.) This letter is followed by a sequence of numbers, such as A*0301, for one allele at the A locus. The first two digits describe the allele type; in most cases the first two digits correspond to the historical serological antigen groupings. *Low resolution* HLA typing refers to alleles which are reported at this two-digit level (e.g., A*03).

The third and fourth digits are used to designate the allele subtypes, wherein alleles are assigned numbers from 01–99 roughly according to their order of discovery. A minimum of four digits thus uniquely defines any allele: by definition, any two alleles which differ in their four-digit number, differ by at least one amino acid. For example, A*0301 and A*0302 do not encode the same protein sequence. Because two-digit names are exhausted after 99 alleles, there are a few oddities in the nomenclature. For example, A*02 and A*92 belong to the same two-digit class as do B*15 and B*95 [14].) See http://www.anthonynolan.org.uk/HIG/lists/nomenlist.html and [2] for more nomenclature details. Sometimes more than four digits are used to designate an allele: the fifth and sixth digits are used to distinguish alleles which differ only by synonymous substitutions (i.e., do not change the amino acid sequence of the protein), while the seventh and eighth digits distinguish alleles which differ in sequence in the non-coding regions of the gene (i.e., the introns or the 5′ or 3′ untranslated regions). For the purpose of our work, we omit this level of detail and limit our analysis to the four-digit level only. In any case, there is not enough data available at the six-to eight- digit resolution level to do any substantial statistical modeling.

Assuming that HLA resolution beyond four digits are ignored, there are still various levels of ambiguity that can arise from molecular (DNA)-based HLA typing methods. For example, rather than knowing unambiguously which two A alleles a person has, one may instead know only a list of possibilities; for example, A*0301-A*3001 or A*0320-A*3001 or A*0326-A*3001. Such *intermediate resolution* types may result from sequence-specific PCR (SSP) based typing where testing with the initial set of PCR primers will yield a list of possible genotypes that a particular person might have (which may require further testing with additional combinations of allele-specific primers and/or cloning and sequencing of clones before an unambiguous type is achieved). As previously mentioned, even modern sequence-based methods may result in ambiguous allele combinations (if sequenced alleles differ outside the genotyped region, or if different possible allele combinations result in the same pattern of observed nucleotide mixtures). Depending on the clinical and/or research purpose of the HLA typing, additional laboratory testing required for achieving high-level (i.e., four-digit) resolution are often not performed for reasons relating to time and cost. In many cases, intermediate-level resolution data are truncated to two-digit resolution; in the previous example, this individual would be reported as having HLA alleles A*03 and A*30.

Although related but different HLA alleles (for example, those alleles which share the same first two digits) sometimes share immunogenic properties, higher resolution data allows for more precise and informative downstream use (e.g., [15]). We are thus motivated to develop low-cost techniques for improving resolution, such as the statistical method introduced here.

The input to our statistical HLA refinement method consists of two data sets. The first is data of interest that have not been typed unambiguously to a four-digit resolution, but for which we would like to increase the resolution as much as possible. The second input is a set of training data consisting of four-digit resolution HLA types for individual people, where the population is drawn from one that is the same (or, in practice, as similar as possible) to the population of interest for which we wish to refine HLA types. First we train our model on the training data. Then we apply this trained model to our limited-resolution data of interest. For example, if a patient in our data set of interest was typed ambiguously at the A locus as having either (1) A*0243, A*0101, or (2) A*0243, A*0122, then our statistical model assigns a probability to each of these two possibilities. More generally, our model assigns a probability to any number of possibilities (not just two), and over many loci. To date, we have used our method, without computational difficulty, to refine up to four loci with 20–130 alleles at each locus, and, on data sets with up to half a million possible haplotypes.

To be precise about what kind of HLA typing ambiguities our approach can tackle, we emphasize that in principle, our approach can handle any kind of ambiguity, so long as that ambiguity has been resolved in the training data set, and so long as the ambiguity can be defined as an allele or set of alleles, taking on some number of clearly defined possibilities. Two common ambiguities that are of interest to researchers are i) *molecular allele ambiguities*, in which we know that one allele, specified unambiguously (e.g., A*02) is actually one of several possibilities (i.e., A*0201, A*0202, A*0203, *etc*), and ii) *genotype ambiguities*, in which ambiguity arising when various combinations of alleles from both chromosomes produce the same patterns of heterozygous nucleotides in the chromatogram). In this paper, we focus our experiments on the first type of ambiguity, although our approach should work on the second kind as well. It may also be of interest to predict high-resolution HLA types from serological data. So long as it is known which serological types map to which molecular types, our model can, in principle, tackle these types of data.

### Related Work

At the core of our HLA typing refinement model is the ability to infer and predict haplotype structure of HLA alleles across multiple loci (from unphased data, since this is the data that is widely available). If certain alleles tend to be inherited together because of linkage disequilibrium between them, then clearly this information can help us to disambiguate HLA types—and far more so than using only the most common allele at any particular locus. We derive a method for disambiguating HLA types from this haplotype model.

Existing methods for haplotype modeling fall into three main categories: *ad hoc* methods, such as Clark's parsimony algorithm [16] which agglomerates haplotypes starting with those uniquely defined by homozygous alleles; EM-based maximum likelihood methods, such as those belonging to the family introduced by Excoffier and Slatkin, and Hawley and Kidd [17],[18], which are related to the so-called *gene-counting* method [19]; and full Bayesian approaches, such as those introduced by Stephens et al. [20], with more recent advances by others (e.g., [21],[22]). Clark's method is no longer used, as it is outperformed by other methods. The full Bayesian methods are more principled than the EM-based methods because they average over all uncertainty including uncertainty about the parameters. However, full Bayesian methods are generally much slower than EM-based methods, and their convergence is generally more difficult to assess [23], making them less attractive for widespread use.

The haplotype modeling part of our approach is most closely related to the EM-based maximum-likelihood methods, although it differs in several crucial respects. To our knowledge, all implementations of EM-based maximum likelihood haplotype models use a full (unconstrained) joint probability distribution over all haplotypes (i.e., over all possible alleles, at all possible loci) with the exception of the partition-ligation algorithms noted below. Furthermore, because they are maximum-likelihood based, they do not smooth the parameter estimates, thereby allowing for unstable (i.e., high variance) estimates of rare haplotypes. Together, these two issues make existing methods difficult to scale to a large number of loci or to a large number of alleles per locus. This scalability problem is widely known (e.g., [17],[24],[25]), and several attempts to alleviate it have been suggested, such as eliminating posterior states which can never have non-zero probability [24], or using a heuristic divide-and-conquer strategy, called partition-ligation [26],[23] in which the joint probability distribution over haplotypes is factored into independent blocks of contiguous loci, and the solutions to each block are then combined. Although these approaches do help alleviate the problems of scalability, the former does so in a fairly minimal way, and the latter places heuristic constraints on the nature of the solution (through use of the blocks). Furthermore, these methods do not address scaling in the number of alleles, which is the larger concern for HLA typing. In addition, these methods do not address the stability of the statistical estimation procedure. Our EM-based approach tackles the issues of scalability by using a parsimonious haplotype parameterization. This especially helps for scaling up to the large number of alleles in HLA data. Our approach also addresses stability by using MAP (*maximum a posteriori*) parameter estimation rather than an ML estimate.

We note that within the HLA community, even recently, haplotype inference seems to be exclusively performed with the most basic EM-based algorithm of Excoffier and Slatkin, and Hawley and Kidd [17],[18] (e.g., [27],[28],[29],[30],[31],[32],[33]). In fact, in one of the most recently available publications, Maiers et al. were unable to perform haplotype inference for more than three HLA loci, resorting to more heuristic techniques beyond this number. With our approach, such limitations are not reached. In addition, as we shall see, our approach is more accurate.

There are two pieces of work which tackle the allele refinement problem using haplotype information: that of Gourraud et al. in the HLA domain [12], and that of Jung et al. in the SNP (single nucleotide polymorphism) domain [34]. Although Gourraud et al. indirectly tackle the HLA refinement problem, their focus is on phasing of HLA data in the presence of ambiguous HLA alleles, and their experimental evaluation is restricted to the phasing task. Additionally, they use the standard, multinomial, EM-based haplotype inference approach, which we show to be inferior for the task of HLA refinement. Also, they do not investigate population-specific effects as we do here. Jung et al., strictly speaking, don't refine their data. Rather, they impute it—that is, they fill in data that is completely missing. The SNP domain is quite different from the HLA domain—the problem of SNP haplotype inference often involves hundreds or thousands of loci, and there are usually only two alleles at each locus (and at most four). HLA haplotype inference, in contrast, involves only a handful of loci with possibly hundreds of alleles at each locus (because we define a locus on an HLA level, not a nucleotide level—although one could do HLA haplotype inference in the nucleotide domain).

Thus, issues of scalability and the specific nature of haplotypic patterns are substantially different between these two domains. With respect to methodology, Jung et al. perform imputation in a sub-optimal way. First, they apply an EM-based haplotype inference algorithm ([23]) to obtain a *single* best phasing of their data (i.e., a ML point estimate). Next, using the statistically phased data, they compute linkage disequilibrium in the inferred haplotypes using the standard measure of Lewontin's linkage disequilibrium. Thus, they ignore the uncertainty over phases which is available from the EM algorithm. Also, they choose only the single best imputed value, ignoring the uncertainty there as well. Our approach incorporates both types of uncertainty. Lastly, the haplotype inference algorithm used by Jung et al. does not account for population-specific effects. Consequently, they do not investigate this area experimentally, as we do here, showing its potential benefits.

One other study touches on statistical HLA refinement [31]. In order to estimate haplotype frequencies on serologically-derived HLA data, Muller et al. modify the standard EM-based haplotype inference approach to be able to use donors with unsplit serological HLA types. However, their main purpose is to estimate haplotype frequencies (at a two-digit serological level) rather than to perform HLA refinement; and their experiments focus on this former task.

## Materials and Methods

Before explaining our model in detail, we first explain the standard EM-based model and training algorithm used for haplotype inference [17],[18]. Without loss of generality, suppose that we are performing haplotype inference over three loci, *l*_1_, *l*_2_, and *l*_3_, with *L^i^* 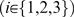 alleles at each locus. Then, in the standard EM-based approach, the probability of a haplotype is parameterized by a multinomial table which gives the probability of every possible haplotype,
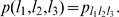
(1)


In this case, there would be 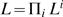 possible haplotypes, requiring *L* parameters, 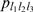. EM is a general algorithm for solving ML/MAP parameter estimates in the presence of missing data/hidden variables [35],[36] (which, here, are the phases). In the present context, EM reduces to iterating between two simple steps:

1. Given the current parameter estimates (for 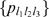), find the distribution of phases for each observed genotype. This is the E-step, where the expectation over haplotypes/hidden states is computed.
2. Given the distribution over haplotypes/hidden states for each observed genotype, compute the maximum likelihood parameter estimates (in this case, the multinomial parameters). This is the M-step, where the parameters are maximized with respect to the *expected complete log likelihood*, where the expectation is taken with respect to the posterior over hidden states, and the complete log likelihood is the likelihood in which the missing information (the phase) has been probabilistically completed proportionally to the posterior distribution over phases.

Note that in both of these steps, it is assumed that the probability of an individual's genotype data having a particular phasing is the product of the probability of each of the two haplotypes defined by the phasing. Thus this approach assumes Hardy-Weinberg equilibrium (HWE).

As mentioned earlier, there are two main problems with this modeling approach. The first is that the number of parameters, *L*, scales badly with the number of loci and with the number of alleles at each locus. This creates two practical problems which quickly come into play —computational limitations on the number of loci/alleles which can be handled by the algorithm [27], and, poor stability with respect to the parameter estimation because the number of parameters tends to be very large relative to the number of data typically available. We alleviate both of these problems using several modifications, and show experimentally the benefits that these modifications provide.

### ASoftmax-Based Haplotype Model

First, we describe a model for *p*(*l*_1_,*l*_2_,*l*_3_,) that uses far fewer parameters than the full table. Using the chain rule of probability, we can write
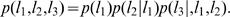
(2)


Equation 2 does not introduce any conditional independencies. If we were to use a (conditional) probability table for each of these three local distributions, then this model would capture exactly the same information as Equation 1 and would not reduce the number of parameters. However, instead of using conditional probability tables, we use softmax regression functions (also known as multilogit regression) [37],[38]. A *softmax regression function* is an extension of logistic regression to more than two target classes. Using a softmax regression function to parameterize 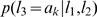, the probability that the allele at the third locus is the *k* ^th^ allele, conditioned on the alleles at the other two loci, *l*_1_, *l*_2_, we have
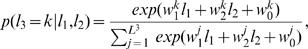
(3)where 
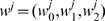
 are parameter vectors of the softmax regression—one for each possible allele, *j*, at the third locus. Thus, the softmax regression function takes a linear combination of the input features, 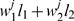, plus a constant term, 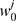, to model each class, which produces a real-valued number for each class. Then, this real-value is exponentiated, and normalized relative to all of the other classes, to yield the probability of interest.

Similarly, the softmax regression function for 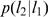 in Equation 2 is written as 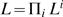
and for *p*(*l*_1_), trivially, as
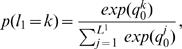
with respective parameters, 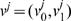 and 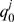. Because the alleles at each locus are discrete in nature, we use a binarized version of the inputs. That is, we use a *one-hot encoding*, wherein each discrete input, *l_i_* = *k* is represented by a binary vector of length *L^i^* that contains all zeros, except at the *k* ^th^ position, which contains a one. Correspondingly, the parameter vectors are augmented in length to match this dimensionality. Thus, in this binary representation, the length of each *w^k^* would be *L* ^1^+*L* ^2^+1, and the total number of scalar parameters required to represent *p*(*l*_1_,*l*_2_,*l*_3_,) would be *M* = *L* ^3^(*L* ^1^+*L* ^2^+1)+*L* ^2^(*L* ^1^+1)+*L* ^1^(1). Note that *M* grows much more slowly here as compared to *L* for the multinomial tables. In particular, *L* grows exponentially in the number of loci and alleles, whereas *M* grows only linearly. Use of full tables versus the softmax regression function relates to the well known bias–variance trade-off [37] which states that the more flexible a model, the more variance one will have in estimating its parameters. To reduce variance, one can decrease the flexibility of the model (as we have done by using softmax regression rather than multinomial parameterizations), thereby increasing the bias of the learned model (because the family of possible models is more restricted). Whether one has chosen a suitable bias-variance trade-off is normally assessed empirically. In the experimental section, we show that the use of the softmax regression function improves the accuracy of the HLA refinement task over use of a multinomial parameterization.

This softmax-based model can be easily extended, by direct analogy, to more than three loci, and far more efficiently than can the multinomial-based model. We note that the additive nature of the softmax regression functions leads to the property that similar haplotypes have similar joint probabilities. Coalescent priors used in some Bayesian approaches also have this property, whereas full tables do not.

### Training the Model with EM

We use the EM algorithm to train our model—that is, to choose good settings of the softmax parameters (*w^j^*, *v^j^*, and *q^j^*) given observed genotype data. The way in which EM operates for our model is very similar to the way in which it works for the multinomial-based models. Again, we iterate between an E-step, where the posterior over possible phases is computed, followed by an M-step, where the parameters of the model are computed based on the posterior computations from the E-step. The difference, of course, is that the posterior uses our softmax model to compute the posterior, and our M-step estimates softmax-regression parameters rather than multinomial parameters.

Formally, let *g^d^* be the observed genotype/HLA data for the *d* ^th^ person in our data set. For example, if we have data for three loci, HLA-A, HLA-B, and HLA-C, then we would have unphased data for each chromosome, for each locus, 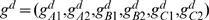. There are 2^number of loci−1^ possible unique phase states, *h_i_^d^*, that this data can take on (assuming no ordering of the chromosomes):
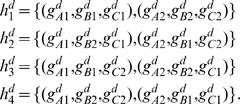



For the E-step, we compute 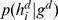 for each data point, for each possible phase. This computation is easily accomplished by determining the likelihood of the data in each possible phase state, and then renormalizing these within each person so that 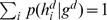. Here, we assume that each phasing is a priori equiprobable. The likelihood of one datum in a particular phase state, *l_i_^d^* is given by the product of the likelihood under our haplotype model, for each of the two chromosomes. For example, the likelihood for the *d* ^th^ genotype to be in phase state 2 is given by
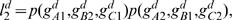
(4)and renormalization of these likelihoods gives us the posterior over phase states for a single individual,
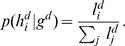



For the M-step, we use the E-step posteriors to compute the parameter estimates. As mentioned, we use MAP parameter estimates which are generally more stable. For the prior distribution of each parameter, we use a zero-centered Gaussian distribution. The use of this parameter prior is sometimes referred to as *L2 smoothing* or *L2 regularization*, because its use is equivalent to adding a penalty term to the log likelihood that consists of the square of the L2 norm of the parameter vectors. Thus, whereas in a maximum likelihood setting we would, in the M-step, maximize the quantity
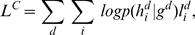
which is the expected complete log likelihood, with respect to the softmax parameters, *w^j^*, *v^j^*, and *q^j^*, we instead maximize the quantity
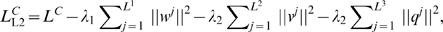
where 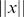 denotes the L2 norm of vector *x*. This quantity is the regularized expected complete log likelihood. The regularization parameters, *λ* = (*λ*_1_,*λ*_2_,*λ*_3_,), which are (inversely) related to the variance of the Gaussian prior, are set empirically using a hold out set. Because this MAP estimation problem is embedded inside of an M-step, the regularization parameters are theoretically not independent (except for *λ*_1_ because it does not depend on the phasing of the data), and hence must be adjusted jointly. We describe how we do so in the experimental section.

The use of other parameters priors is possible. One commonly used alternative is the Laplacian prior or, equivalently, L1 regularization. In experiments not reported here, we have found L2 and L1 regularization to provide comparable performance on our task.

By iterating between the E-step and the M-step from some chosen parameter initialization (or, some posterior initialization), we are guaranteed to locally maximize the log posterior of the data, *L*, (keeping the *λ_j_* fixed),
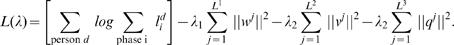



We note that one can smooth/regularize the parameters of the multinomial table using a Dirichlet prior. This smoothing has the effect of adding pseudo-counts to the observed counts of the data when computing the ML estimate during the M-step. In our experiments, we compare our model against both the traditional multinomial haplotype model and a Dirichlet regularized multinomial model.

The ML (and L2-regularized MAP) softmax regression parameter estimation problem within a single M-step is a convex problem, and hence not subject to local minima. In contrast, *L*(*λ*) is not convex due to unobserved phase and is subject to local minima. Nonetheless, in our experiments, we did not find local minima to be a large problem, and leave further discussion of this to the Experimental section.

As with the traditional algorithm used in the HLA community, our EM algorithm assumes random mating. In the discussion, we propose one way to remove this assumption.

### Using the Model for Statistical HLA Refinement

As discussed, we first train our model using the EM algorithm on a data set consisting of four-digit resolution HLA data from a population similar to that of our data of interest. We then use the model to probabilistically refine our lower-resolution data set. To do so, we refine each person's HLA type independently of the others. The way we do so, is to exhaustively write out a list of all possible unique four-digit phasings that are consistent with each person's observed genotype data. We do so by first writing out all possible (mixed resolution) phases, and then expanding each of these to all possible four-digit phases. For example, if one person's observed genotype in the data set of interest was 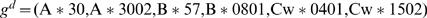, then we obtain
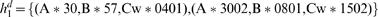
(5)

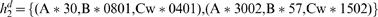
(6)

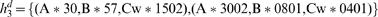
(7)

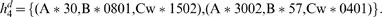
(8)


Expanding Equation 5, for example, we then obtain,
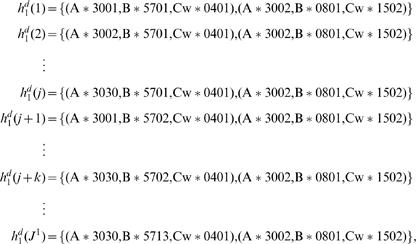



Similarly, we expand each of Equations 6–8 to obtain an additional *J* ^2^, *J* ^3^, and *J* ^4^ possible four-digit phasings. The total number of possible four-digit phasings consistent with this person's observed genotype is thus *J* = *J* ^1^+*J* ^2^+*J* ^3^+*J* ^4^. Alternatively, if our data set of interest contains genotype-ambiguity (in the form of possible pairs of alleles), then we expand the data in all possible ways consistent with those pairs.

If our desired endpoint is a statistical estimate of phased four-digit data, then we need only compute and renormalize the likelihood of each member of the list (to get the posterior probability of each pair of four-digit haplotypes). However, usually we are interested in a probability distribution over the possible four-digit genotypes. To obtain this distribution, we sum the posterior probabilities of those members of the list that are consistent with each observed genotype. For example, 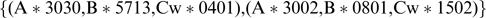 and 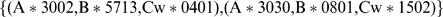 would give rise to the same observed genotype: (A*3030, A*3002, B*5713, B*0801, Cw*0401, Cw*1502), and so their posterior probabilities would be summed together (along with any other entries in the list which mapped to the same observed genotype) to obtain the posterior probability of that genotype.

### Leveraged Population Models

Because haplotype patterns are often population (ethnicity)-specific, a natural approach is to use separate models for each population, when the populations are known. For example, if the low-resolution data of interest pertained primarily to individuals of European descent, then one would train a model using data from a European population. Or, if the low-resolution data consisted of both European and Amerindian populations, then one would train a model on European and Amerindian populations separately, and then refine the data of interest using the appropriate model.

Nonetheless, it is likely that some haplotype patterns are population-specific whereas others are not, or far less so. Consequently, it would be useful to combine data across populations, so that as much data as possible is available for parameter estimation. The challenge of course is how to combine data when appropriate, to maintain population-specific training data when appropriate, and to make good choices automatically. One way to achieve this goal is to augment the feature space (which so far consists of binary encodings of HLA alleles) with population features. We can, for example, include a one-hot encoding of the population labels in our features. Alternatively or in addition, we can add features that correspond to conjunctions of the one-hot encodings of allele and population label. Whereas the first type of augmentation, which we refer to as *simple*, allows us to weight the importance of a haplotype by a linear combination of populations, the second type of augmentation, which we call *conjunctive*, allows us to model specific haplotype–population interactions. In the evaluation section, we shall see that such *leveraged population models* can improve performance. Furthermore, we shall see that the first type of augmentation provides a winning effect over training populations separately and that adding the second type of augmentation leads to no additional improvement in the data set examined.

The idea of leveraging information across multiple populations is closely related to some of our previous work on epitope prediction in which we show how to leverage information across HLA alleles [39], and is an instance of what is sometimes called multi-task learning [40]. Xing et al. use a hierarchical Bayesian model to achieve a similar approach when inferring SNP haplotypes [22].

### Why Require an Independent Training Data Set?

One could imagine using a mixed-resolution data set of interest (which contains some four-digit HLA types) as its own training data since EM naturally handles incomplete data. If the data that are missing four-digit resolution information are *ignorable*, then such an approach is straightforward [41]. By definition, data that are ignorable have the property that the probability that a particular datum is missing (in this case, does not have a four-digit HLA type) is independent of the true, underlying value of the missing datum (in this case, the four-digit HLA type). Of course, if the data are not ignorable, then such a procedure can produce large errors. Unfortunately, missing high-resolution HLA data are not likely to be ignorable, and hence we require an independent data set with no missing data.

### Statistical Significance

To assess statistical significance of the difference of the performance of two models (e.g., softmax compared to multinomial), either in terms of the number of correct MAP predictions, or, in terms of the test log likelihood, we used a non-parametric, permutation-based, paired test, wherein the null hypothesis is that the average of the pair wise difference in scores is zero.

Suppose the test set contains *D* individuals, 1,,*D*, and that each model, *m*, assigns a score, 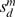, to each individual (where again, this score is either the log probability of the correct assignment, or the number of correct MAP predictions). Then to compare two models, *m*_1_ and *m*_2_, we do the following:

- Compute the average difference between paired scores, in each algorithm, 
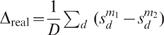
.
- For permutation, *k* = 1,,*K* (we use *K* = 10,000), permute the data in a pair wise fashion to obtain data from the null distribution, and then compute the average difference between paired scores in this permuted data. That is, for each permutation, k,
-
  - For each datum, *d*, swap the value of 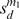 with 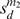 with probability one half. Call the resulting permuted data vectors, 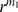 with 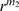, which are the permuted equivalents of 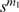 with 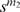.
  - Compute the average difference between paired, permuted scores,
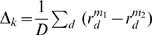
.
- Then the two-sided p-value for method *m*_1_ being statistically different from *m*_2_ is given by the proportion of times that the average difference observed on permutated data matched, or exceeded that observed on the real data. Formally,
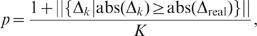

- where 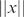 denotes the size of the set *x*, and abs(*x*) denotes the absolute value of *x*. The addition of one to the numerator smoothes the estimate of *p* so as to take into account the number of random permutations performed. Without this smoothing, one could easily achieve results of *p* = 0 by using too few random permutations. This induces a conservative bias (reducing the type I error, and increasing type II error), which diminishes as the number of permutations increases.

### Data Sets

We used data sets from two main sources, and denote the number of individuals in each by N. The first data set is a collection of private data derived from a large collection of disease cohorts and controls that were all typed in the laboratory of Mary Carrington. This data set comprises data from four populations, across three loci, as summarized in Table 1. Note that most of the African data are derived from African-American individuals, with a small proportion from outside the United States (N = 776). The Hispanic and European data are solely US-based, while the Asian data originated in Asia. Because alleles C17, C18 and A74 were almost never fully resolved to four digits in this data set, we left these as two digit designations. All but 0.1% of HLA alleles in the private data set represented *common and well-defined alleles* (as classified in [42]). Because these large data sets comprise numerous smaller data sets (and sub-populations), we tested each data set, at each locus, for deviance from Hardy-Weinberg Proportions (HWP) using the conventional MCMC approximation to the exact test [43]. The number of MCMC samples was chosen to ensure that the estimated p-value was within 0.01 of the true one with 99% confidence. Alleles deviating from HWE at a level p = 0.1 or stronger (lower p-values) were: European HLA-C locus (p = 0.003), African HLA-C (p = 0.0001), Asian HLA-A, -B, C (p = 0, p = 0, p = 0.0004). In all of these cases, except for the Asian HLA-C locus, the deviation was toward homozygosity. EM algorithms for haplotype frequency estimation have been shown to be robust against deviations toward homozygosity, with the explanation that increased homozygosity reduces the amount of missing phase information that the EM algorithm must overcome [25]. In any case, our experimental results demonstrate that this issue is not of such great concern as to invalidate our approach.

**Table 1:** Summary of Private Data

ethnicity	N	# unique A alleles	# unique B alleles	# unique C alleles
North American European	7526	81	129	48
North American African	3545	60	106	42
Asian	1318	43	76	30
North American Hispanic	881	47	106	35

Class I genotyping: Genomic DNA was amplified using locus-specific primers flanking exons 2 and 3. The PCR products were blotted on nylon membranes and hybridized with a panel of sequence-specific oligonucleotide (SS0) probes (see http://www.ihwg.org/protocols/protocol.htm). Alleles were assigned by the reaction patterns of the SSO probes. Ambiguous SSOP typing results were resolved by sequencing analysis. Only exons 2 and 3 were examined during HLA typing. Any subtypes determined by sequences outside these exons were not distinguished. In these cases the earliest recognized alleles were assigned, normally the ones of the smallest digit in their names (e.g., B*5801 instead of B*5811).

The second data set was taken from the publically available dbMHC database (http://www.ncbi.nlm.nih.gov/mhc/), which we used to test our population-augmented model [44],[45],[46],[47],[48], and also for use of our model on four-loci data [49]. These data are summarized in Table 2.

**Table 2:** Summary of dbMHC Data

ethnicity	N	# unique A alleles	# unique B alleles	# unique C alleles	# unique DRB1 alleles
Irish	1000	26	49	23	33
North American Asian	393	34	66	24	NA
North American European	287	28	48	21	NA
North American Black	251	28	49	23	NA
North American Hispanic	240	35	62	25	NA
North American Amerindian	229	27	55	22	NA
All except Irish	1400	48	102	31	NA

## Results

In order to evaluate our model, and also to compare how it performs to a multinomial-based model, we use data sets consisting of four-digit resolution HLA data from individuals. Then we synthetically mask the known four-digit allele designation for some loci and some individuals, at random. In this way, ground truth is available for quantitative assessment. Specifically, we use the following set-up:

1. Start with a four-digit HLA resolution data set, *D*.
2. Randomly partition *D* into 80% for training (*D*_train_) and 20% for testing (*D*_test_).
3. To learn good settings of the regularization parameters, randomly partition *D*_train_ into 80% for a regularization training set (*S*_train_) and 20% for a regularization hold out set (*S*_hold_). Train a model on *S*_train_, for each value of the regularization parameters, and then test its performance on *S*_hold_. Select the regularization parameters which perform best.
4. Using the best regularization parameters, train the model on *D*_train_, and then test its performance on *D*_test_).

To test the performance as mentioned above, we randomly mask 30% of the four-digit HLA types (on an individual and independent allele basis) in the test/hold-out set. That is, we truncate the last two digits of their four-digit designation. We then use our HLA refinement to obtain a probability distribution for all four-digit HLA types which are consistent with the masked values. Then we assess the prediction in two ways. One, we take the four-digit type with the highest probability as the single, best answer, and then count how many of these are correct. We refer to this criterion as the *percentage of correct MAP predictions*. Two, we compute the log probability of the correct four-digit resolution HLA type under our predictive distribution. We refer to this as the *test log likelihood.* If we divide this quantity by the number of masked alleles and then exponentiate, we obtain the geometric mean probability of the correct four-digit allele under our learned model (which is more intuitive than the test log likelihood). We refer to this criterion as the *geometric mean probability.* The first criterion (% correct MAP) is intuitive but informal and coarse. It allows us to easily get a handle on the performance, but throws away valuable information concerning the probabilities generated by the model which may be useful in downstream analyses of the data. In our experiments, we report performance according to both types of criteria. Note that these values should be compared only within a given test set.

Although we mask the HLA types at random, this is likely not the same process that is responsible for the true, observed, experimental process that results in masking. Nonetheless, we feel that it is a reasonable proxy, because it focuses on how well haplotype patterns have been learned, how strong these patterns are, and how much they can be used to refine HLA data, which is the question of interest. Additionally, we measure performance under a 100% masking, and also a locus-by-locus masking, for broader testing of the performance of our model.

In addition to experimenting with our softmax-based model, and the multinomial (with and without regularization), we also compare performance to a baseline model of allele marginals. In this baseline model, the probability over four-digit HLA types is proportional to the frequency of that allele in the training set, regardless of the HLA data at other loci. This model, by construction, cannot capture haplotype structure. As we shall see, this model does not perform well.

For the softmax-based model, we first learned the best value for *λ*_1_ (i.e., for the first locus) since it is independent of the others. Then, fixing the value of *λ*_1_ at its best value, we set all other *λ_i_* = 0.1. For each of the other loci, *i*, one at a time, we next found the best value of *λ_i_* conditioned on the fixed values of the other regularization parameters. We iterated through the loci in this manner until no changes were made. In our experiments, this process reached convergence after only two or three cycles through the loci, indicating that, in practice, the parameters 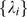 are largely independent of one another. We optimized a single parameter by searching a grid of possible values. The grid used in our softmax-based model experiments was 50;10;5;1;0.5;0.1;0.05;0.01;0.001. For the multinomial-based model, we used the grid 1;5;10;50;100;500;1000;5000;10,000;50,000 for the equivalent sample size of the Dirichlet distributions.

Lastly, to determine if there is a statistically significant difference between our methods (in terms of either test log likelihood, or number of correct MAP predictions), we use a permutation-based, non-parametric, paired test in which the null hypothesis is that the average of the pair wise difference in scores is zero. Because 10,000 permutations were used, the smallest p-value that could be obtained was 
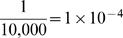
.

### EM Sensitivity to Initialization

Because the objective function we use, the penalized likelihood, is not convex, our parameter estimation and hence HLA refinement can be sensitive to the initial parameter setting. (Note that by parameters, we mean *w^j^*, *v^j^*, and *q^j^* within the multi-logit functions, and not the regularization parameters, *λ_i_*, nor the phasings, *h_i_*.) To assess the sensitivity of performance to the initial parameters, we initialized the parameters randomly between 0 and 1 five different times. We performed this assessment on our Hispanic-labeled private data because this set corresponds to one of the smaller ethnicity-specific data sets, and because this ethnic label is less well defined than others. Both factors (small data sets, and ethnicities that are not well-defined) tend to produce greater sensitivity to parameter initialization.

When training our softmax-based model, the geometric mean probability across the five initializations was aways 0.5255. (A larger geometric mean probability is better.) In all five runs, 262 of the 306 masked alleles were correctly predicted, indicating little sensitivity to parameter initialization. Similarly, for the regularized multinomial-based model, the geometric mean probabilities across the five initializations was always 0.4180. In all five runs, 262 of the 306 masked alleles were correctly predicted, again indicating little sensitivity. For the unregularized multinomial-based model, the geometric mean probabilities across the five initializations were: 0.0077, 0.0117, 0.0126, 0.0092, and 0.0105. Of the 306 masked alleles, 260, 265, 260, 266, and 262 were correctly predicted across the five runs, indicating a far greater sensitivity to initial parameters.

The geometric mean probability was best for the softmax-based model, followed by the regularized multinomial, followed by the unregularized multinomial model (which does poorly due to its inability to make stable estimates for the huge number of parameters it requires). This is a pattern we shall see throughout our experiments.

The sensitivity we see here will allow us to gauge how important observed differences are in the remainder of the experiments, where we always initialize the parameters to be all zero. Of course, when deploying this method in a real setting, it would be wise to try several parameter initializations, and then to choose the one that yields the highest likelihoods on hold-out data. Also note that, for the unregularized multinomial model, we regularize it with an equivalent sample size of 1×10^−16^ so that negative infinities do not appear when haplotypes not seen in the training sample appear in the test set.

### Large Scale Data Set Comparison

**Figure 1:**
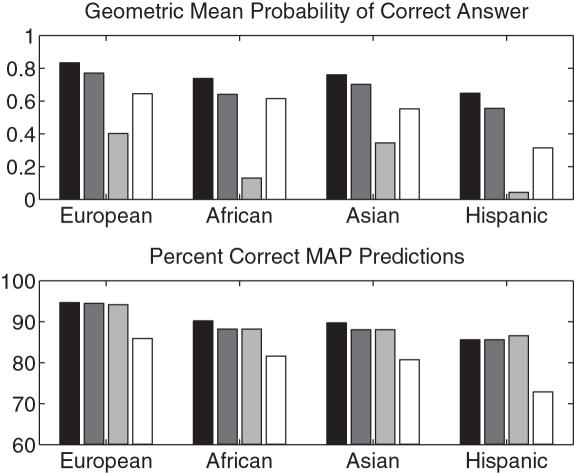
Results on private data, separately for each ethnicity. Each set of grouped bars represents the four different modeling approaches. From darkest to lightest: softmax, regularized multinomial, unregularized multinomial, allele marginal. The number of masked alleles, respectively, in the European, African, Asian, and Hispanic data sets was 2669, 1287, 477, and 306, respectively.

Next we used our large, private data set to measure the refinement performance of the various models we have discussed. We trained and tested within each ethnic population separately. The results are summarized in Figure 1.

The softmax model has the best performance overall and can correctly resolve a substantial number of ambiguous alleles. In terms of both criteria, the softmax model is significantly better than the other methods (see Table 3 for p-values). The allele marginal model consistently has the worst performance in terms of number of correct MAP predictions, presumably because it does not make use of linkage disequilibrium. In contrast, it significantly outperforms the unregularized multinomial model in test log likelihood (*p* = 1×10^−4^), because the allele marginals are naturally regularized due to the small number of parameters.

**Table 3:** Statistical Significance Results on Private Data, Separately for Each Ethnicity.

Method 1	Method 2	log likelihood p-value	# correct MAP p-value
softmax*	regularized mult.	p = 10^−4^	p = 2.8×10^−3^
softmax*	non-reg. mult.	p = 10^−4^	p = 8×10^−4^
softmax*	allele marginals	p = 10^−4^	p = 10^−4^
regularized mult.*	non-reg. mult.	p = 10^−4^	p = 0.51
non-reg. mult.*	allele marginals*	p = 10^−4^	p = 10^−4^

*: Denotes the method that performed better (except for the last row, where the allele marginals perform better than the unregularized multinomial on the log likelihood, but worse on the number of correct MAP predictions.

### When Training and Test Set Are Not Identically Distributed

In realistic settings where our algorithm will be deployed, it is likely that the data set of interest is not drawn from exactly the same distribution as the training data. To get a sense of how robust our approach is to deviations from this idealized setting, we have performed several experiments more closely mimicking a realistic setting. In particular, we evaluated our refinement accuracy when the training and test distributions were drawn from different populations.

First, we split the dbMHC Irish data set (HLA-A, HLA-B, HLA-C alleles) into 80% training data and 20% test and masked 30% of the test alleles to two digits. Then we trained a model using the training data, and tested on the test data. Next, we used the model we had previously trained on the ‘private North American European’ data, and used this model to predict the same masked, Irish alleles. Of the 200 people in the Irish test set, there was one person who contained one allele never observed in the European data (B*2409, which is actually a null allele, B*2409N, for which the typing of the private data was not capable of finding). After removal of this person, we then compared the performance when using the dbMHC Irish data set itself for traning, as compared to using our much broader private European data set for training. The resulting test geometric mean probabilities of the test set were 0.8851 when training with the dbMHC Irish, and 0.8891 with the private European. This difference was not significant (p = 0.44).

Next, we used the model trained on the private Asian data to predict a 30% masking of 279 dbMHC Canton Chinese individuals [50] with HLA-A,-B, -C data (we randomly chose this population among the Asian dbMHC populations available). Nine of these individuals had alleles not appearing in the training data (A*0210, B*1505, B*1803, B*3508, B*3520, B*4010, B*5801, B*7802), and after their removal, we achieved a prediction accuracy of 441/487 = 91%, roughly equal to the 90% achieved when testing on the private

Asian data set itself. Because this dbMHC data set was not large enough to partition into a training and test set, we were not able to measure accuracy achieved when training on itself. This is true for the next three dbMHC data sets as well, in which we perform similar experimentation.

Next we used a model trained on the private North American African data set, to predict masked alleles in 251 dbMHC African American individuals, of which five individuals contained alleles not matching the training data (A*6804, B*1502, B*1515, B*5802). After removal of these individuals, 321/373 = 86% of masked alleles were correctly predicted, which is lower than the 90% accuracy achieved when testing on the private North American African data itself. Results were comparable when we first removed individuals from Africa from the training data (leaving only US-based individuals of Africans descent).

Next, we used a model trained on the private North American European data set (containig 776 individuals), to predict masked alleles in 287 dbMHC North American European individuals, of which three individuals contained alleles not matching the training data (B*1802, B*4408, B*5202). After removal of these individuals, 478/510 = 94% of masked alleles were correctly predicted, roughly equal to the 95% accuracy achieved when testing on the private North American European data set itself.

Finally, we used a model trained on the private North American Hispanic data set, to predict masked alleles in 240 dbMHC North American Hispanic individuals, of which 13 individuals contained alleles not matching the training data (A*0212, A*0213, A*2422, A*2608, A*3401, A*6805, B*5105, B*3509, B*4406). After removal of these individuals, 344/400 = 86% of masked alleles were correctly predicted, comparable to accuracy achieved when testing on the private North American Hispanic data set itself.

Based on this small set of experiments, we believe it may often be feasible to use our broadly defined ethnic categories for resolving ambiguity in other, independently created data sets falling in to the same broad category, or falling into a much more specific sub-category. Of course, this may not generally be true, and in particular, it may be less true for African-derived data. Additionally, a user of a trained model might have access to some high-resolution data for their population of interest, and could thus see how well the trained model works for the subset of their data (by synthetically masking it) before using the model to resolve ambiguity in their low-resolution data.

Note that there are two statistical *desiderata* when using our method: 1) to use a training data set which mostly closely mimicks the HLA haplotype distribution of the data set of interest, and 2) to get as many training data as possible. Critically, these two desiderata are frequently odds with one another. That is, often a data set of interest is sub-population specific and therefore difficult to obtain high resolution data for in large quanitities. However, by loosening the strictness of the match between training and test populatations, one can often significantly increase the amount of data available. Without more data and experimentation, it is diffult to assess the optimal trade-off between these desiderata. However, as we see, using broad, even presumably admixed training data, can lead to useful results.

### Sensitivity to Training Data Set Size

**Figure 2:**
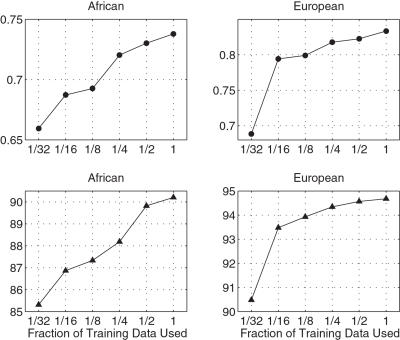
Sensitivity to training data set size for the European and African data sets. Top row shows the geometric mean probabilities; the bottom row shows the percentage of correct MAP predictions.

To determine whether the availability of more training data may lead to improved refinements, we examined the sensitivity of performance to the size of the training set. For the European and the African private data sets, we iteratively halved the sample size of training data, where the largest available training data set sizes were, respectively, 6020 and 2836. The results shown in Figure 2 suggest that more training data would improve the performance on the African data set, and to a smaller extent, on the European data set. Note that the African data set is smaller to start with than the European one, and also known to be more genetically diverse; both are explanations for the observed trends.

### Leveraged Population Models

**Figure 3:**
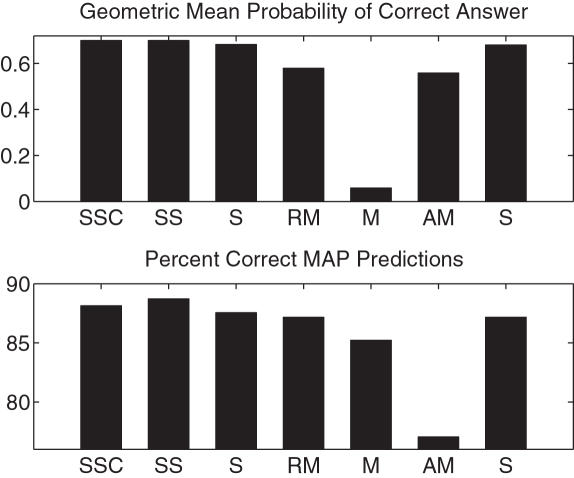
Results for population-augmented model. Abbreviations are: SSC = softmax+simple+conjunctive, SS = softmax+simple, S = simple, RM = regularized multinomial, M = non-regularized multinomial, AM = allele marginals, S = separate. The number of masked alleles in the test set was 514. For all methods, except ‘separate’, a single model was trained on data from all ethnicities.

To determine whether leveraging information across populations is useful, we compared our leveraged population models to those built separately on each population. We did so on data from dbMHC, which contains a diverse set of populations. (We excluded the Irish population because this population is extremely homogeneous relative to the others.) Recall that we introduced two types of leveraging features: simple and conjunctive. We used our softmax model both with the simple features alone, and with both the simple and the conjunctive features, as shown in Figure 3.

The performance of the population-augmented models are significantly better than the softmax model on test log likelihood (e.g., *p* = 0.02 when comparing softmax+simple to softmax). Although ethnicity labels are notoriously unreliable, they clearly provide beneficial information here. Also, the addition of conjunctive features lends to no apparent improvement.

### Sensitivity to Variable Ordering

**Figure 4:**
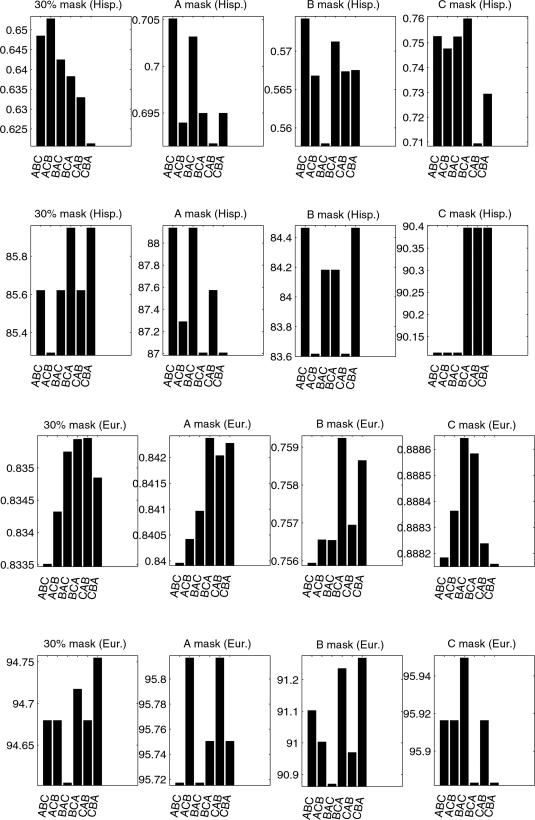
Sensitivity to Variable Ordering. Top two rows are for the Hispanic data set; bottom two rows are for the European data set. Within each of these, the top row is the geometric mean probabilities, and the bottom row shows the percent correct MAP predictions. The number of masked alleles, respectively, in the Hispanic 30% and loci masks (A,B,C), was 306 and 354. The number of masked alleles, respectively, in the European 30% and loci masks (A,B,C), was 2669 and 3012.

Because we use softmax regression functions in our haplotype model, the order in which we apply the chain rule (Equation 2) to our loci will have an effect on predictive accuracy. We examined the sensitivity of performance to variable ordering on three loci (A,B,C) using the European and Hispanic data sets. The results are shown in Figure 4 in which a locus order of ‘B A C’ means we used 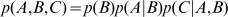. The experiments labeled ‘30% mask' denote the performance using the 30% random masking procedure we used in our earlier experiments. Additionally, we systematically masked all (and only) A alleles (‘A mask’), and separately, all and only B alleles (‘B mask’), and all and only C alleles (‘C mask’). This procedure allows us to see if the variable ordering differentially affects our ability to predict particular loci. Statistical significance was measured only on the difference in test log likelihoods.

For the ‘30% mask’ experiments, no statistically significant (*p*⩽0.01) differences were found between variable orderings (and hence the results of our previous experiments should not have been effected by this issue). For the locus-specific maskings in the European data set, only the B alleles showed significant differences (order 1 vs. 4, p = 0.0002; 1 vs. 6, p = 0.001; 2 vs. 4, p = 0.003; 2 vs. 6, p = 0.004; 3 vs. 4, p = 0.0006; 4 vs. 5, p = 0.006). For the locus-specific maskings in the Hispanic data set, the A alleles showed some significant differences (order 1 vs. 2, p = 0.004; 1 vs. 5, p = 0.001; 3 vs. 5, p = 0.004), the B alleles did not show any, and the C alleles showed one (order 4 vs. 5, p = 0.003).

Note that it is possible to use a parsimonious model which is not dependent upon variable ordering (a so-called ‘undirected’ model [51] in the parlance of the graphical models community). In particular, one can form pair-wise ‘compatibility’ functions between all pairs of HLA loci so that
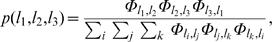
where the 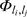 are scalar parameters of the model and where the sum in the denominator is a normalizing constant and sums over all possible haplotypes, (*l_i_*,*l_j_*,*l_k_*,). However, brief experimentation of this model applied to the current problem did not indicate increased performance relative to our softmax-based model.

### Locus-Specific Predictive Accuracy

**Figure 5:**
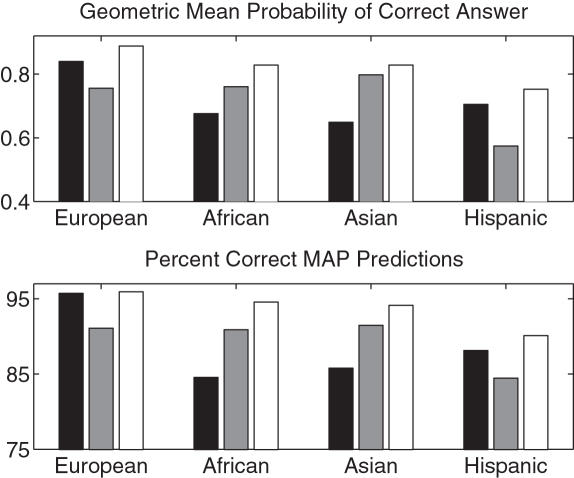
Locus-Specific Predictive Accuracy. Each set of grouped bars, from darkest to lightest, represents, respectively, A-masking, B-masking, and C-masking. The number of masked alleles in each masking was 3012, 1418, 528, and 354, respectively, for the European, African, Asian, and Hispanic test sets.

In some domains, the ability to predict certain loci is of greater importance than others. For example, in HIV research, the ability to predict B alleles is often paramount (e.g., [15]). We measured locus-specific prediction accuracy for each locus by applying locus-by-locus masking to all four populations in the private data. Figure 5 shows the results, which do not indicate any particular pattern. Note that the number of possible alleles at each locus has a direct effect on our ability to predict (as does the linkage between one locus and the others), and so we might expect, *a priori*, for the B alleles to be more difficult to predict, although this does not appear to be the case.

### Low Resolution Prediction

**Figure 6:**
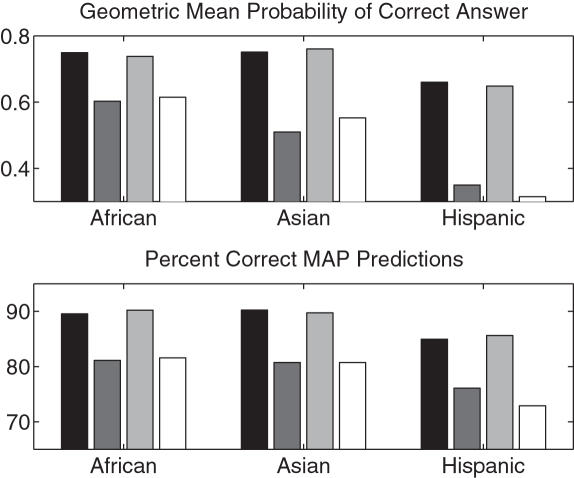
Low Resolution Prediction. Each set of grouped bars represents, from darkest to lightest, respectively, 100% mask with softmax model, 100% mask with allele marginals model, 30% mask with softmax model, 30% mask with allele marginals model. The number of masked alleles for the 100% mask was 4254, 1429, and 1062, and for the 30% mask, 1287, 477, and 306, in the African, Asian, and Hispanic test sets, respectively.

Finally, in some instances, only low-resolution data (i.e., two-digit resolution) is available. Consequently, we investigated the prediction accuracy of our algorithm in this situation—that is, when 100% of the alleles were masked to two-digit. The results for the private African, Asian, and Hispanic data sets are shown in Figure 6. Because of the large number of allele combinations in the European data set, it was not possible to perform this experiment in a reasonable amount of time using the current sequential implementation of the algorithm. This problem should not be a big concern, however, as the algorithm can be easily parallelized.

In order to gauge how much haplotype information is being used in this context, we compare the results to those from the allele marginal model. In all cases, the softmax model performs significantly better than the allele marginal model (*p* = 1×10^−4^ for all three population comparisons on the test log likelihood). Thus, a large amount of haplotype information is being used by our model in this 100% masking context, and prediction of four-digits from strictly two-digit data is feasible. For comparison, Figure 6 includes the results presented earlier from the 30% masking experiments. To make the test log likelihoods comparable, we have normalized them by the number of alleles in the test set. Interestingly, the performance is comparable across the different maskings according to both criteria.

### Four-Loci Example/Class I and Class II

**Figure 7:**
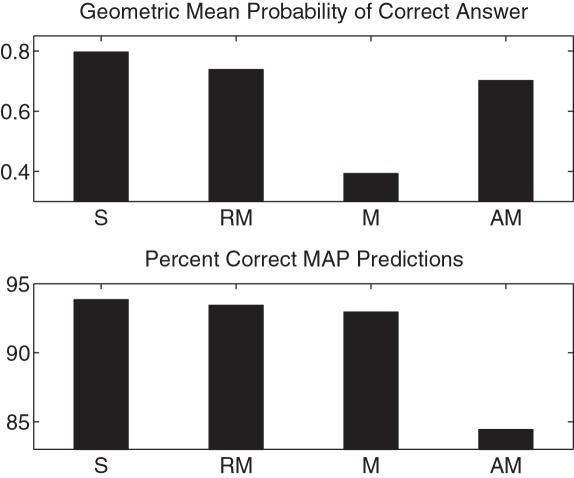
Four-loci, dbMHC Irish data. Abbreviations are: S = softmax, RM = regularized multinomial, M = non-regularized multinomial, AM = allele marginals. A total of 468 alleles were masked in the test set.

We compared our methods on data with four loci, spanning the HLA-A, -B, -C and -DRB1 loci. The four-loci data available to us, with the largest sample size, was the Irish set in dbMHC. As shown in Figure 7, we see that the relative performance of the methods is roughly the same as in earlier experiments. Given that LD may not be as strong between class I and class II alleles, it is of interest to determine how well each locus can be predicted. Thus we used a locus-specific masking, as described earlier. The accuracy at each of the HLA-A, -B, -C and -DRB1 alleles was respectively 97%, 98%,99%, and 80%. This indicates that there is not sufficient linkage between the HLA-A, -B, -C loci and the HLA -DRB1 locus to accurately resolve ambiguity at the DRB1 locus. However, it may be the case that with additional class II loci, refinement of class II data would be feasible.

## Discussion

We have introduced a method for statistical refinement of low or intermediate resolution HLA data, when a full resolution training data set from a similar population is available. In doing so, we have also improved upon the EM-based approach to haplotype estimation by using a more parsimonious parameterization of the haplotype distribution. Experimentally, we show both that it is feasible to use statistical approaches for HLA refinement, and also that our method outperforms the standard multinomial-based models used throughout the HLA community for haplotype estimation. Our HLA refinement method helps to mitigate the limiting factor of cost in HLA typing today, and allows for lower/intermediate resolution, or historical data to be statistically refined when it cannot be refined by assay. A tool based on our approach is available for research purposes at http://microsoft.com/science.

Although there is widespread caution about the use of assigned, or self-defined ethnicity labels [52], we show that the labels associated with dbMHC data carry useful information. Furthermore, we show that by augmenting our softmax-based HLA model, we can make use of these labels to increase the amount of data available while automatically using it in a population-appropriate manner. Future work of interest would be to model the data in yet another way: using a *mixture of haplotype models*, in which each component of the mixture represents one well-defined population (either as defined in the training data, or as uncovered in an unsupervised manner). Then, when data contain multiple populations without ethnicity labels or when labeled populations contain mixtures of latent (unknown) subpopulations, one can use these mixture models to uncover population structure and appropriate weightings of the different populations for individuals in a data set of interest.

Because our modeling approach assumes that the training and testing populations are drawn from the same distribution, one should take care when trying to use this approach for case-control studies where case and controls are thought to be drawn from different distributions. One may also be wary of using this approach in the domain of transplantation, for similar reasons (patients requiring transplants likely make up a specific sub-population). However, since HLA ambiguity resolution is applied in the area of transplants to potential donors in a registry, rather than the patients themselves (who are routinely typed at high resolution), application in this domain should not be problematic.

As with the traditional algorithm used in the HLA community, our EM algorithm assumes HWE. One could make a small change to our model which would allow us to circumvent making such an assumption. In the models discussed so far, the probability of data in a particular phasing is defined as follows. If a haplotype, *h*, is specified by partitioning the genoytpes, *g_A_*_1_,*g_B_*_1,_ *g_C_*_1,_ *g_A_*_2_,*g_B_*_2,_ *g_C_*_2_ into two sets: 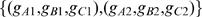, then the probability of the data given this phasing is defined as the product of the probability of each haplotype:
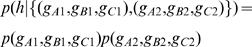
(9)where each of the probabilities *p*(*g_A_*_1_,*g_B_*_1_,*g_C_*_1_) and *p*(*g_A_*_2_,*g_B_*_2_,*g_C_*_2_) are specified by a *haplotype model* (e.g., softmax or multinomial). To circumvent the assumption of HWE, one could instead define a model which does not factor this probability into two independent terms:
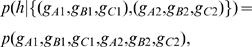
(10)where now we would not have a haplotype-based model, but instead a more generic, *ordered-genotype* model, which could itself be given a softmax-based parsimonious parameterization. The downside of such an approach is that we essentially halve the amount of available data, because we no longer have two independent data samples from each individual, and hence far more data would be required to effectively make use of such a model.

Future work in probabilistic HLA refinement may involve comparing EM-based approaches to full Bayesian approaches. Also, an interesting, though perhaps computationally difficult avenue to pursue would be the use of HLA DNA sequences to better model rare haplotypes, or the use of SNP data to directly predict HLA types.
